# Model Development for Risk Assessment of Driving on Freeway under Rainy Weather Conditions

**DOI:** 10.1371/journal.pone.0149442

**Published:** 2016-02-19

**Authors:** Xiaonan Cai, Chen Wang, Shengdi Chen, Jian Lu

**Affiliations:** 1 Transportation Research Center, School of Naval Architecture, Ocean and Civil Engineering, Shanghai Jiao Tong University, Shanghai, P.R. China; 2 School of Transportation Engineering, Tongji University, Shanghai, P.R. China; 3 College of Transport and Communications, Shanghai Maritime University, Shanghai, P.R. China; Chongqing University, CHINA

## Abstract

Rainy weather conditions could result in significantly negative impacts on driving on freeways. However, due to lack of enough historical data and monitoring facilities, many regions are not able to establish reliable risk assessment models to identify such impacts. Given the situation, this paper provides an alternative solution where the procedure of risk assessment is developed based on drivers’ subjective questionnaire and its performance is validated by using actual crash data. First, an ordered logit model was developed, based on questionnaire data collected from Freeway G15 in China, to estimate the relationship between drivers’ perceived risk and factors, including vehicle type, rain intensity, traffic volume, and location. Then, weighted driving risk for different conditions was obtained by the model, and further divided into four levels of early warning (specified by colors) using a rank order cluster analysis. After that, a risk matrix was established to determine which warning color should be disseminated to drivers, given a specific condition. Finally, to validate the proposed procedure, actual crash data from Freeway G15 were compared with the safety prediction based on the risk matrix. The results show that the risk matrix obtained in the study is able to predict driving risk consistent with actual safety implications, under rainy weather conditions.

## Introduction

Weather affects driver capabilities, vehicle performance, pavement friction, roadway infrastructure, and crash risk through visibility impairments, precipitation, high winds, and temperature extremes. According to the National Highway Traffic Safety Administration in the United States, from 2002 to 2012, there were on average 1,311,970 weather-related crashes each year, resulting in 480,338 injuries and 6,253 deaths and the vast majority of weather-related crashes happened on wet pavements (74%) and during rainfall (46%) [1]. Similarly in China, based on an annual report of road accidents statistics, there were about 56,809 weather-related crashes in 2012, resulting in 65,243 injuries and 17,040 deaths and over forty percent of these crashes occurred under rainy weather conditions [2]. Whether it is in the United State or China, rainy weather has significantly negative impacts on driving safety. Therefore, it could be necessary to develop travel weather warning systems (TWWS) to help drivers identifying driving risk on rainy days.

Road Weather Information System (RWIS) as a kind of TWWS has been successfully applied for aiding decision-making of highway administrations in Europe and America. This system can collect and monitor real-time traffic and weather conditions, and disseminate early warning information to drivers. The cores of the RWIS are their risk estimate models, which were developed based on large weather-related crash data. However, most developing countries are unable to establish their own TWWS due to the lack of sufficient and reliable weather-related crash data. For example, in China, there is only one meteorological criterion [3] used for road weather management instead of the professional TWWS, and the criterion only considers weather parameters and ignores significant impacts of traffic conditions such as vehicle type and traffic volume.

To fill the gap, this paper attempts to develop a procedure of risk assessment used to evaluate driving risk on rainy days. And the procedure should include combined impacts of multiple factors instead of only meteorological factors and not depend much on historical weather-related crash data.

## Literature Review

Generally, there are three types of research methods on evaluating rain-related driving risks. Firstly, their engineering aspects are well understood. In particular, the physical effects of rain on pavement friction [4,5] and driver visibility [6,7] were given much attention. However, converting estimates of frictional change or impaired visibility into driving risk is much more difficult considering the stochastic nature of crashes.

Secondly, many traffic safety research activities related to rainy weather focus on analyzing accidents. Palutikof found that rainy weather was the most significant one among all weather factors which resulted in traffic fatalities [8]. In a study by Sherretz and Farhar, it was found that there existed a linear positive correlation between rainfall and traffic crash frequency [9]. More detailed findings about impacts of rainy weather on traffic safety were summarized by Andrey et al. [10] and Eisenberg [11]. However, few researches in developing countries (e.g. China) have been identified due to lack of rain-related crash data.

Thirdly, risk perceptions by drivers are used to identify rain-related driving risks. Andrey and Knapper found that drivers did recognize the evaluated risk associated with adverse weather conditions [12]. Some studies showed that drivers’ subjective assessments of the relative danger associated with driving during various weather scenarios were reasonably consistent with collision studies [12,13]. That is to say, subjective data from drivers could be applied for evaluating driving risk on rainy days. Driving inexperience is one of the key predictors of crash rates [14], with young novice drivers being at risk particularly [15]. Furthermore, the higher accident rate of young drivers is due to their poor cognitive skills [16] and inattention [17]. On the contrary, experienced drivers can adapt their strategies in time by anticipating various demands of different driving conditions [18]. In light of these, if driving perception by experienced drivers could be provided to young novice drivers, it could be expected that they would better identify driving risk on rainy days and lower crash potential.

Risk assessment is a scientific process of evaluating adverse effects caused by a substance, activity, lifestyle, or natural phenomenon [19]. According to Berdica, the definition of accident risk consisted of two parts: the probability of accident occurrence and the consequence [20]. Some studies [21] considered Relative Risk Ratio (RRR) as an effective method to quantify crash risk under adverse weather conditions. But the method requires a large number of weather-related accident records to match pairs. Another alternative method is using a risk matrix [20], which includes combined effects of consequence and probability.

The study summarized in this paper attempts to develop a procedure for risk assessment of driving on the freeway under rainy weather conditions, which is based on drivers’ risk perception. Data were derived from drivers’ subjective questionnaire and actual traffic crashes on China National Freeway G15 (with kilometer post: k1184+275~k1215+870). A risk matrix with the combined effects of consequence and probability was established to determine levels of driving risk. Furthermore, actual crash data on the same freeway segment from May in 2008 to June in 2011 were used to examine the validity of the risk matrix. Being warned by the level of driving risk, drivers, especially young novice drivers, could better identify surrounding risk.

## Methods

The study was approved by the Ethics Committee of Shanghai Jiao Tong University. The data derived from drivers’ questionnaire drafted by Dr. Xiaonan Cai was analyzed anonymously and therefore no additional informed consent was required. In addition, Dr. Xiaonan Cai and other researchers in Transportation Research Center including Chen Wang, and Jian Lu administered this questionnaire. Moreover, the details about the questionnaire can be seen in the section of Data.

Ordered logit models and rank order cluster analysis are the two main mathematical models in this study. The former was used to calculate impacts and corresponding probabilities associated with various driving circumstances on rainy days. The latter was employed for driving risk classification.

### Ordered Logit Model

The dependent variable, level of impact on driving (*C*_*i*_), is considered discrete in the modeling with *C*_*i*_ ranged from slight impact, general impact, serious impact, and catastrophic impact, and *i* representing the *i*^*th*^ driver. Observable independent variables include vehicle type, rain intensity, traffic volume, and location. As a general practice, a non-observable variable *ε*_*i*_ is assumed to fit a logistic distribution in order to calculate a continuous latent variable *C*_i_^*^, which is called as impact on driving, or

$$C_{i}^{*}=\sum_{j}^{J}x_{ij}\beta_{j}+\varepsilon_{i},\;C_{i}=1,2,\ldots ,M$$ (1)

where, *J* is number of observable independent variables, *M* is number of dependent variable, *β*_*j*_ is the coefficient for the *j*^*th*^ variable, and the dependent variable *C*_*i*_ has the following relationship with the latent variable *C*_i_^*^:

$$C_{i}=\left\{ \begin{array}{ll} 1 & \;if\;C_{i}^{*}\leq c_{1}\\ 2 & \;if\;c_{1}<C_{i}^{*}\leq c_{2}\\ 3 & \;if\;c_{2}<C_{i}^{*}\leq c_{3}\\ \vdots & \;\vdots \\ M & \;if\;c_{M-1}<C_{i}^{*} \end{array}\right.$$ (2)

where, *c*_*k*_ (*k* = 1, 2, …, *M*-1) are threshold values to satisfy: *c*_1_< *c*_2_ <…<*c*_M-1_. As mentioned previously, the ordered logit model sets non-observable variables to fit logistics distribution. Thus, probabilities of different levels of impact can be calculated as follows:

$$\left\{ \begin{array}{l} P(C_{i}=1)=\frac{\text{exp}\left(c_{1}-\left(\sum x_{ij}\beta_{j}\right)\right)}{1+\text{exp}\left(c_{1}-\left(\sum x_{ij}\beta_{j}\right)\right)}\\ P(C_{i}=2)=\frac{\text{exp}\left(c_{2}-\left(\sum x_{ij}\beta_{j}\right)\right)}{1+\text{exp}\left(c_{2}-\left(\sum x_{ij}\beta_{j}\right)\right)}-\frac{\text{exp}\left(c_{1}-\left(\sum x_{ij}\beta_{j}\right)\right)}{1+\text{exp}\left(c_{1}-\left(\sum x_{ij}\beta_{j}\right)\right)}\\ \ldots \ldots \\ P(C_{i}=M)=1-\frac{\text{exp}\left(c_{M-1}-\left(\sum x_{ij}\beta_{j}\right)\right)}{1+\text{exp}\left(c_{M-1}-\left(\sum x_{ij}\beta_{j}\right)\right)} \end{array}\right.$$ (3)

By definition, driving risk (*R*_*i*_) represents impact on driving (*C*_i_^*^) multiplied by the corresponding probability (*P*_*i*_). Consequently, a series of *R*_*i*_ can be calculated to measure driving risk on rainy days.

### Rank Order Cluster Analysis

It is assumed that driving risk (*R*_*i*_) could be obtained and sorted by the ascending order, as indicated by *R*_(1)_, *R*_(2)_, …, *R*_(n)_. A certain category (*G*), including *R*_(i)_, *R*_(i+1)_, …, *R*_(j)_ and satisfying *j*>*i*, can be expressed as G = {*i*, *i*+1, …, *j*}. On the basis, the diameter of *G*, *D* (*i*, *j*), is calculated as follows:

$$D\left(i,j\right)=\sum_{t=i}^{j}{\left(R_{\left(t\right)}-R_{G}\right)}^{2}$$ (4)

where, *R*_*G*_ is the mean value of driving risk in the category *G*.

When driving risk is divided into *k* categories, different categories are expressed as follows:

$$\left\{ \begin{array}{l} G_{1}=\left\{i_{1},i_{1}+1,\ldots ,i_{2}-1\right\}\\ G_{2}=\left\{i_{2},i_{2}+1,\ldots ,i_{3}-1\right\}\\ \ldots \ldots \\ G_{k}=\left\{i_{k},i_{k}+1,\ldots ,i_{k+1}-1\right\} \end{array}\right.$$ (5)

where, *i* is to satisfy 1 = *i*_1_< *i*_2_ <…< *i*_*k*_ < *i*_k+1_ *= n*+1.

The loss function is defined by the formula (6), which represents the sum of diameters for *k* categories. When *n* and *k* are given, the smaller the loss function is, the better the classification of driving risk is. Then the minimal loss function has a recursion relationship shown in the formula (7).

$$L\left[b\left(n,k\right)\right]=\sum_{t=1}^{k}D\left(i_{t},i_{t+1}-1\right)$$ (6)

$$\left\{ \begin{array}{l} L\left[P\left(n,2\right)\right]=\underset{2\leq j\leq n}{\text{min}}\left\{D\left(1,j-1\right)+D\left(j,n\right)\right\}\\ L\left[P\left(n,k\right)\right]=\underset{k\leq j\leq n}{\text{min}}\left\{L\left[P\left(j-1,k-1\right)\right]+D\left(j,n\right)\right\} \end{array}\right.$$ (7)

where, *b* (*n*, *k*) represents a kind of classification method; *P* (*n*, *k*) represents an approach to minimize the loss function. That is to say, when *n* and *k* are given, *P* (*n*, *k*) represents the optimal categories of driving risk.

In this study, driving risk is classified into four categories (*k* = 4). Thus, the optimal categories of driving risk can be determined as the following method. Firstly, *j*_*4*_ that minimizes the formula (8) should be found. Then, the fourth category is expressed as *G*_4_ = {*j*_4,_ *j*_*4*_+1,…, *n*}.

$$L\left[P\left(n,4\right)\right]=L\left[P\left(j_{4}-1,3\right)\right]+D\left(j_{4},n\right)$$ (8)

Secondly, *j*_*3*_ should be found, satisfying the formula (9). Then, the third category is expressed as *G*_3_ = {*j*_3,_ *j*_*3*_+1,…, *j*_*4*_-1}.

$$L\left[P\left(j_{4}-1,3\right)\right]=L\left[P\left(j_{3}-1,2\right)\right]+D\left(j_{3},j_{4}-1\right)$$ (9)

The rest of categories can be found in the same manner. Therefore, {*G*_1,_ *G*_2,_ *G*_3,_ *G*_4,_} are the optimal categories of driving risk.

## Data

### Questionnaire Design

**Fig 1:**
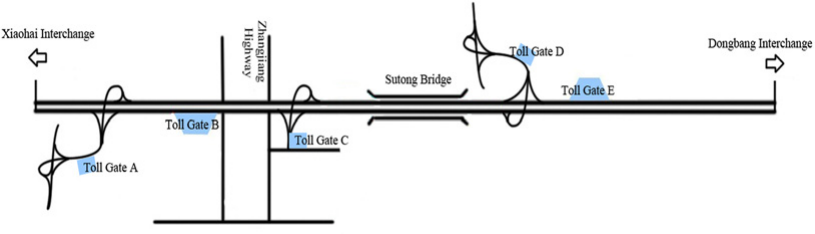
A general view of the segment of National Freeway G15 (Kilometer post: k1184+275~k1215+870).

The segment of National Freeway G15 is located between Suzhou city and Nantong city in Jiangsu Province, with about 32 km in length and a six-lane in both directions. And a general view of the segment is shown in Fig 1. Since the segment opened to traffic for only a few years, it could not accumulate enough crash data to be used for risk assessment of driving on rainy days. And crash data derived from other similar freeways in China could not be obtained, given possibly political impacts. Thus, a questionnaire to drivers was designed for collecting drivers’ risk perception on rainy days. In addition, some crash records collected from the segment were used to validate the proposed procedure of risk assessment.

The questionnaire survey collects information of driver, vehicle, rain intensity, and traffic condition. In order to help drivers better understand the survey, some explanations have been noted. First, according to the national specification JTG B01-2003 [22], two-axle large trucks and multi-axle large trucks are combined into a category of large vehicles. Thus, vehicle type only consists of small vehicles (including middle-size vehicles) and large vehicles.

**Fig 2:**
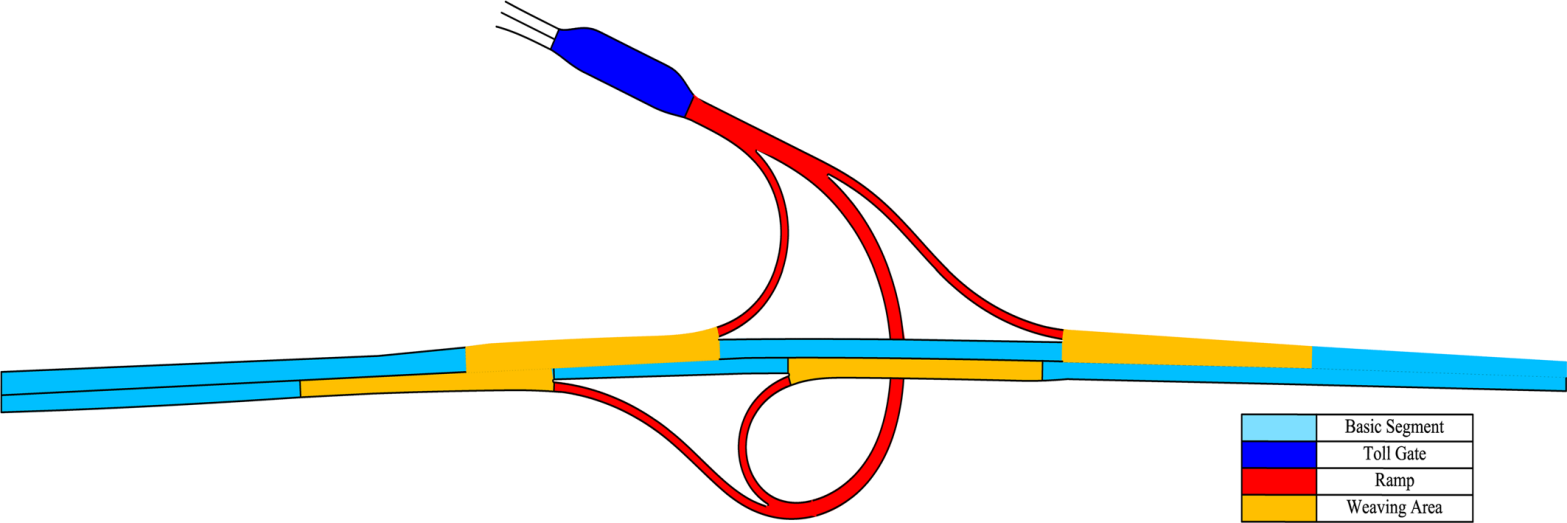
Basic segment, toll gate, ramp, and weaving area around the Toll Gate D.

Second, the freeway segment is divided into four parts, including basic segments, toll gates, ramps, and weaving areas. For example, as shown in Fig 2, the four parts around the Toll Gate D are labeled by four different colors. Specifically, the toll gate is the segment between two change points of roadway width around the Toll Gate D; the weaving areas indicate the segments that are 500 meters from converging or diverging points on the mainline; the ramps are the connecting segments between the toll gate and the weaving areas; the basic segments are the remaining segments except for the weaving areas on the mainline.

Third, the category descriptions of rain intensity and traffic volume are summarized in Table 1. In the group of rain intensity, the visibility is categorized into four levels based on the specification QX/T 111–2010 [3] and the definitions of light rain, moderate rain, heavy rain and rain storm in this study are consistent with those in the weather forecast, which are easier for drivers to understand. In addition, the traffic volume is categorized into four levels based on the specification JTG B01-2003 [22]. For the freeway segment, actual volume data had been being monitored and collected by the G15 Highway Management System (HMS) from May in 2008 to June in 2011. Furthermore, in Table 1, *V* indicates actual data of hourly volume in one direction when the questionnaire survey is made, and *C* indicates the maximum of hourly volume in one direction from May in 2008 to June in 2011.

**Table 1:** Category descriptions of rain intensity and traffic volume.

**Levels of Rain Intensity**	
**Levels**	**Descriptions**
I	visibility about 500 meters, light rain
II	visibility about 200 meters, moderate rain
III	visibility about 100 meters, heavy rain
IV	visibility less than 50 meters, rain storm
**Levels of Traffic Volume**	
**Levels**	***V*/*C***
I	0.00~0.31
II	0.31~0.67
III	0.67~0.86
IV	0.86~1.00

### Data Collection

Questionnaire surveys were prepared to acquire the data of drivers’ demographic information and risk perceptions. Demographic information included age, gender, the type of vehicle driven, and driving experience. Drivers were asked to identify the risks (i.e. impacts) of different driving conditions on a four-point scale (slight, general, serious, catastrophic).

Surveys were conducted at sites including Toll Gate B, Toll Gate E and a service area around Toll Gate B in Fig 1. The duration of surveys was about three months from April to June in 2012 during which rainy weather were expected. With the help of law enforcement officials, respondents were randomly selected according to the last digit of their license plates. Meanwhile, one-on-one questionnaire surveys were employed from 7:00 a.m. to 11:00 a.m. and from 2:00 p.m. to 6:00 p.m. Each questionnaire took about 13 minutes on average. The total number of questionnaires received in this survey was 1694, and the number of effective questionnaires (driving experience more than two year, no illegal crash records in the past year and the number of trips on the freeway each month not less than two) among them was 1216.

The descriptive statistics of the main variables derived from drivers’ questionnaires and actual volume data are summarized in Table 2. In addition, a crosstab of rain intensity and impact on driving was developed. The coefficients of Spearman correlation and Fisher’s exact test were calculated, which is equal to 0.675 and 797.374 (sig. <0.05), respectively, indicating a significantly positive relationship between them.

**Table 2:** Descriptive statistics of the main variables.

**Continuous Variables**				
**Variable Name**	**Mean**	**Standard Deviation**	**Minimum**	**Maximum**
Driver’s Age	36.31	6.64	26	55
Driving Experience	11.25	5.32	3	20
Number of Trips on the Freeway Each Month	5.22	2.58	2	10
**Categorical Variables**				
**Variable Name**	**Classification**	**Frequency**	**Percent %**	**Cumulative Percent %**
Gender	Male	724	59.5	59.5
	Female	492	40.5	100
Vehicle Type	Small Vehicle	709	58.3	58.3
	Large Vehicle	507	41.7	100
Rain Intensity	I	303	24.9	24.9
	II	303	24.9	49.8
	III	305	25.1	74.9
	IV	305	25.1	100
Location	Basic Segment	402	33.1	33.1
	Toll Gate	406	33.4	66.5
	Ramp	245	20.1	86.6
	Weaving Area	163	13.4	100
Traffic Volume	I	273	22.4	22.4
	II	333	27.4	49.8
	III	366	30.1	79.9
	IV	244	20.1	100
Level of Impact	Slight Impact	220	18.1	18.1
	General Impact	350	28.8	46.9
	Serious Impact	375	30.8	77.7
	Catastrophic Impact	271	22.3	100

## Results

### Assumptions

The study assumes that perceived risk by drivers is consistent with actual crash statistics in evaluating driving risk under rainy weather conditions. Past studies show that drivers’ subjective assessment of relative dangerousness associated with driving during various weather scenarios are reasonably consistent with collision-based studies [12,13]. But it is undeniable that risk perception could be subject to variation among drivers and regions. Thus, crash data on rainy days were used to validate whether the procedure of risk assessment based on drivers’ risk perception was feasible (in section of Crash Data Validation).

### The Ordered Logit Model

The study specifies four important factors from the survey, including vehicle type, rain intensity, traffic volume, and location. Symbols and definitions of the variables are listed in Table 3. Among them, three factors, except location, are defined as ordinal variables. The factor of location is considered as a nominal variable with four categories, and defined as three dummy variables (0, 1). With the initial variable settings, the first ordered logit model used for risk assessment is not well fitted, for the variables of “traffic volume” and “location on the freeway” are not significant at the degree of confidence of 0.95 (AIC = 1318.541, SC = 1357.790). Therefore, some adjustments on independent variables should be made (the second model with smaller AIC and SC values associated with the first model, see in Table 4. First, “traffic volume” is redefined to have two categories (i.e. low volume and high volume). Second, for “location on the freeway”, ramp and weaving area are combined into one category. That is, “location on the freeway” is defined as two dummy variables.

**Table 3:** Symbols and definitions of variables in the ordered logit model.

Variable and Symbol	Definition	Frequency
Vehicle Type	*x*_*1*_ = 1, Small Vehicle	709
*x*_*1*_	*x*_*1*_ = 2, Large Vehicle	507
Rain Intensity	*x*_*2*_ = 1, Level of Rain Intensity = I	303
*x*_*2*_	*x*_*2*_ = 2, Level of Rain Intensity = II	303
	*x*_*2*_ = 3, Level of Rain Intensity = III	305
	*x*_*2*_ = 4, Level of Rain Intensity = IV	305
Location	(*x*_*3*_, *x*_*4*_) = (0, 0), Roadway	402
*x*_*3*_, *x*_*4*_	(*x*_*3*_, *x*_*4*_) = (1, 0), Toll Gate	407
	(*x*_*3*_, *x*_*4*_) = (0, 1), Ramp and Weaving Area	408
Traffic Volume	*x*_*5*_ = 1, Low Volume (Level of Traffic Volume = I, II)	606
*x*_*5*_	*x*_*5*_ = 2, High Volume (Level of Traffic Volume = III, IV)	610
Level of Impact	*C* = 1, Slight Impact	220
*C*	*C* = 2, General Impact	350
	*C* = 3, Serious Impact	375
	*C* = 4, Catastrophic Impact	271

**Table 4:** Results of the ordered logit model.

**Score Test for the Proportional Odds Assumption**					
**Chi-Square**		**DF**		***Pr* > ChiSq**	
12.5820		10		0.2480	
**Testing Global Null Hypothesis: BETA = 0**					
**Test**		**Chi-Square**		***Pr* > ChiSq**	
Likelihood Ratio		381.8495		< .0001	
Score		278.8282		< .0001	
Wald		278.8418		< .0001	
**Model Fit Statistics**					
**Criterion**		**Intercept Only**		**Intercept and Covariates**	
AIC		1573.346		1201.497	
SC		1586.404		1236.318	
-2 Log L		1567.346		1185.497	
**Goodness-of-Fit Statistics**					
**Criterion**		**Value**		***Pr* > ChiSq**	
Deviance		120.3138		0.8288	
Pearson		125.7924		0.7239	
R-Square		0.4859		NA	
**Analysis of Maximum Likelihood Estimates**					
**Parameter**		**Estimate**	**Standard Error**	**Wald Chi-Square**	***Pr* > ChiSq**
Vehicle Type	*x*_*1*_	1.0460	0.1914	29.8619	< .0001
Rain Intensity	*x*_*2*_	1.5078	0.0952	251.0182	< .0001
Location	*x*_*3*_	0.7817	0.2012	15.0892	0.0001
	*x*_*4*_	1.5011	0.2074	52.3833	< .0001
Traffic Volume	*x*_*5*_	0.8592	0.1653	27.0064	< .0001
Intercept4		-9.5106	0.5817	267.3385	< .0001
Intercept3		-7.3720	0.5224	199.1591	< .0001
Intercept2		-5.1258	0.4693	119.3206	< .0001

The second ordered logit model was estimated using SAS software and model results are listed in Table 4. Firstly, the *Pr* value of score test for the proportional odds assumption is equal to 0.2480, meaning the null hypothesis (i.e. the ordered logit coefficients are equal across the levels of the outcome) could not be rejected. Secondly, *Pr* values of statistics of testing global null hypothesis are less than 0.05, meaning there is at least one that could not be equal to zero among all ordered logit coefficients. Thirdly, in term of model fit statistics, criterion AIC and SC provide a means for model selection and promising models give small values for these criteria. Finally, the R-square of the model is equal to 0.4859. With linear regression different, when R-square values of ordered logit models are more than 0.3, the models would be considered to a well fit.

In addition, considering association of predicted probabilities and observed responses, the percent of concordant pairs is equal to 81.3% and *c* statistic is equal to 0.827. In conclusion, the ordered logit model has good quality to describe drivers’ risk perception of driving on the freeway under rainy weather conditions.

Meanwhile, coefficients of independent variables and model intercepts were estimated using maximum likelihood values. As presented in Table 4, each independent variable is statistically significant and has a positive relationship with the dependent variable (i.e. level of impact). Impact (C^*^) and level of impact (*C*) have a following relationship with all parameters (variables) specified in Table 4:

$$\begin{array}{l} C^{*}=1.0460x_{1}+1.5078x_{2}+0.7817x_{3}+1.5011x_{4}+0.8592x_{5}\\ C=\left\{ \begin{array}{l} 1\;if\;C^{*}\leq 5.1258\\ 2\;if\;5.1258<C^{*}\leq 7.3720\\ 3\;if\;7.3720<C^{*}\leq 9.5106\\ 4\;if\;9.5106<C^{*} \end{array}\right. \end{array}$$ (10)

As a consequence, the probabilities (*P*_*i*_) of four levels of impact (*C*_*i*_) could be calculated as follows:

$$\left\{ \begin{array}{l} \text{logit}\left(P_{4}\right)=-9.5106+1.0460x_{1}+1.5078x_{2}+0.7817x_{3}+1.5011x_{4}+0.8592x_{5}\\ \text{logit}\left(P_{3}+P_{4}\right)=-7.3720+1.0460x_{1}+1.5078x_{2}+0.7817x_{3}+1.5011x_{4}+0.8592x_{5}\\ \text{logit}\left(P_{2}+P_{3}+P_{4}\right)=-5.1258+1.0460x_{1}+1.5078x_{2}+0.7817x_{3}+1.5011x_{4}+0.8592x_{5}\\ P_{1}=1-\left(P_{2}+P_{3}+P_{4}\right) \end{array}\right.$$ (11)

where, *P*_*1*_, *P*_*2*_, *P*_*3*_, *P*_*4*_ is the probability of slight impact, general impact, serious impact, and catastrophic impact, respectively.

### Weighted Driving Risk

By definition, driving risk (*R*) equals impact on driving (C^*^) multiplied by the corresponding probability (*P*). However, it should be noted that the definition of driving risk literally commensurate adverse events of high impacts and low probabilities with events of low impacts and high probabilities. An effective solution for such a problem is to assign a weight variable to each individual level of impact. Thus, weighted driving risk (*WR*) can be expressed as follows:

$$WR=C^{*}\cdot P\cdot W=\left(1.0460x_{1}+1.5078x_{2}+0.7817x_{3}+1.5011x_{4}+0.8592x_{5}\right)\cdot P\cdot W$$ (12)

where, *P* and *W* are the probabilities and the weights for four levels of impact, respectively.

The weight variable was determined by Delphi method with focus group discussions. Ten members with experience and expertise in traffic safety and operations joined the discussions. The average values of weights from the ten experts for each level of impact are used as the final weights, which are equal to 0.6, 0.9, 1.2, and 1.5 for slight impact, general impact, serious impact, and catastrophic impact, respectively. Based on the formula (12), a series of *WR* can be calculated and the larger the *WR* value, the higher perceived risk by drivers. In other words, according to the *WR* values, driving risk under various conditions can be measured and compared. However, *WR* is only a relative measure of driving risk without any physical implications, which is not easily understood by drivers and road weather managers. Thus, these *WR* values should be classified into different levels of early warning. In addition, sensitivity analysis of weights is presented in section of Discussions.

### The Rank Order Cluster Analysis

**Fig 3:**
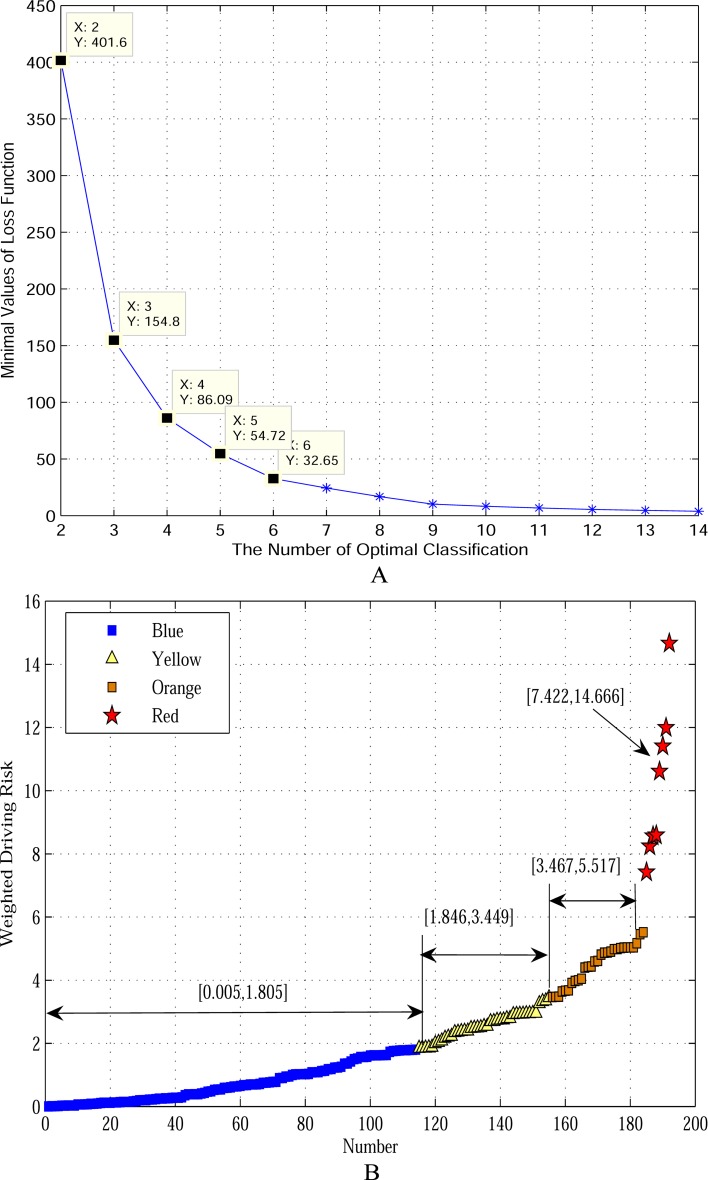
Results of the rank order cluster analysis through MATLAB programming. Panel A shows minimal loss functions vary with number of classifications. Panel B shows distribution ranges of four levels of weighted driving risk (k = 4).

As stated previously, this study classified weighted driving risk (*WR*) using the rank order cluster analysis. Firstly, the *WR* values were sorted by the ascending order. Secondly, the minimal loss functions for different categories were calculated by the MATLAB programming and the results show that the minimal loss functions decrease with number of classifications increasing in Fig 3A. In China, an early warning system normally has four different warning colors. Accordingly, the *WR* was classified into four levels (*k* = 4) and the corresponding loss function was equal to 86.09. Finally, the optimal classification of *WR* was found and the four levels of *WR* were coded as blue, yellow, orange, and red, with red color representing the highest risk. Furthermore, the corresponding ranges for four warning colors are shown in Fig 3B. That is, when a *WR* value lies in the range from 0.005 to 1.805, a blue early warning is disseminated. However, such method used for early warning could be too complex in real practice. A feasible way is to establish a risk matrix with combined effects of impact and probability.

### Risk Matrix

**Fig 4:**
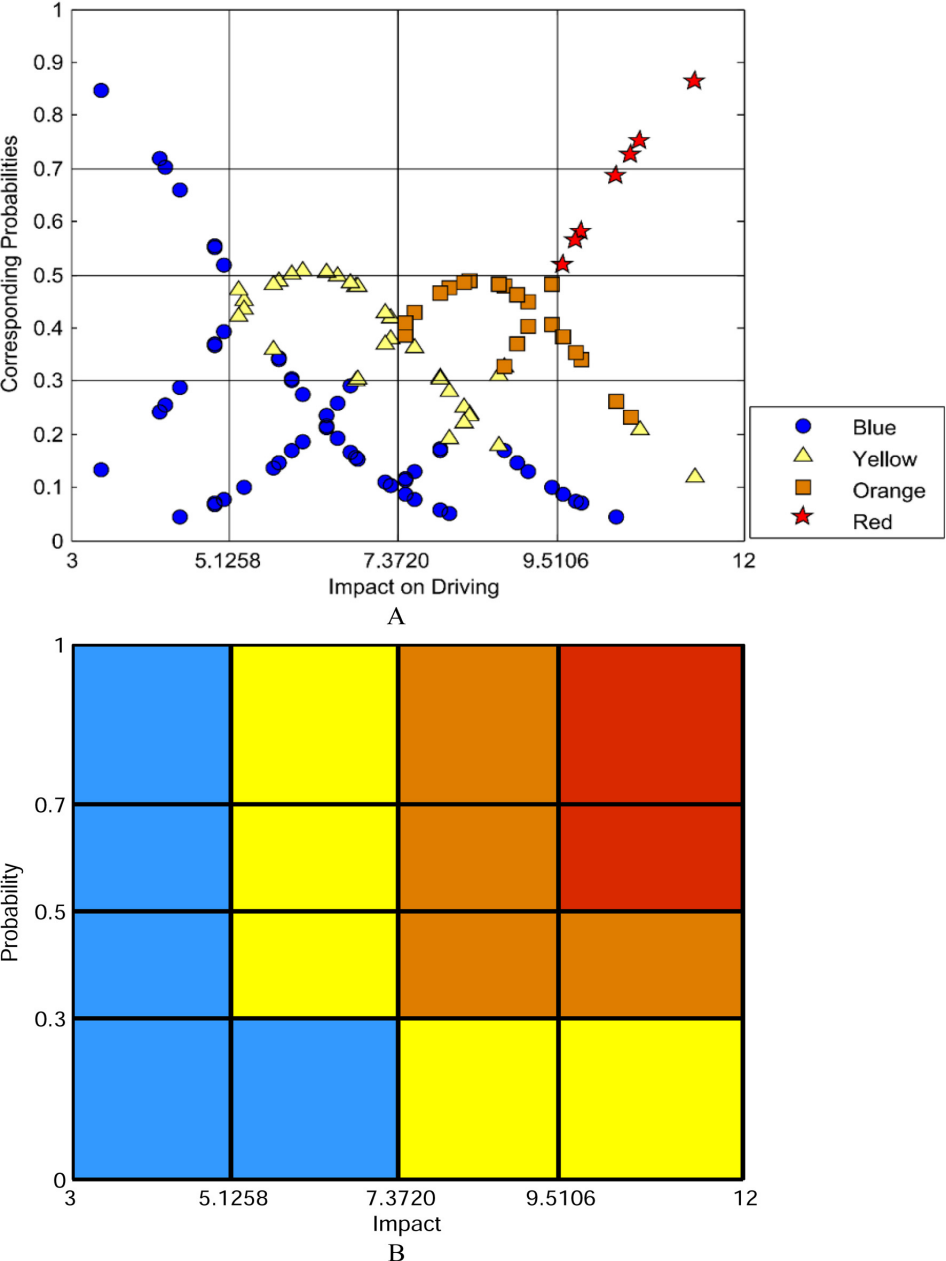
Conducting the risk matrix of driving on freeway under rainy weather conditions. Panel A shows distribution of early warning colors of weighted driving risk.Panel B shows risk matrix with combined effects of impact and probability.

To develop a risk matrix, it is most important to determine thresholds of impact and probability. According to the formula (12), Fig 3B was transformed into Fig 4A, with impact as the abscissa and probability as the ordinate. According to Fig 4(*A*), the thresholds of probability were set to be 0.3, 0.5, and 0.7 subjectively. Meanwhile, three thresholds of impact in the ordered logit model were equal to 5.1258, 7.3720, and 9.5106, respectively. Based on the colors of *WR* in Fig 4A, the risk matrix was developed as shown in Fig 4B. Thus, a specific driving condition on rainy days can be determined a warning color, by matching its impact and probability with the matrix.

## Crash Data Validation

To validate the proposed risk matrix, 708 crashes derived from the segment of National Freeway G15 (K1184+275~K1215+870) were collected from May in 2008 to June in 2011. Crash data from the segment are available with specific information related to crash characteristics, meteorological elements and traffic conditions. Crash severity was divided into four categories: slight crash, general crash, serious crash, and fatal crash with the definitions based on: (1) damage to traffic facilities, (2) injury to occupant, (3) occupancy to lane. Crash descriptions are listed in Table 5.

**Table 5:** Descriptions of crashes from the segment of National Freeway G15.

Variables Title	Definition	Frequency	Percent %	Cumulative percent %
Crash Tim2e	00:01~04:00	43	6.1	6.1
	04:01~08:00	88	12.4	18.5
	08:01~12:00	151	21.3	39.8
	12:01~16:00	176	24.9	64.7
	16:01~20:00	166	23.4	88.1
	20:01~24:00	84	11.9	100
Crash Location	Roadway	112	15.8	15.8
	Toll Gate	353	49.9	65.7
	Ramp and Weaving Area	243	34.3	100
Precipitation Type	Rain	323	45.6	45.6
	*Rain Intensity I*	278	39.3	39.3
	*Rain Intensity II*	25	3.5	42.8
	*Rain Intensity III*	11	1.5	44.3
	*Rain Intensity IV*	9	1.3	45.6
	Non-Rain	385	54.4	100
Crash Vehicle Type	Small Vehicle	658	76.9	76.9
	Large Vehicle	198	23.1	100
Crash Type	Fixed-object Crash	251	35.5	35.5
	Rear-end Crash	248	35.0	70.5
	Side Crash	151	21.3	91.8
	Others	58	8.2	100
Traffic volume (*V*/*C*)	≤ 0.67	221	31.2	31.2
	> 0.67	487	68.8	100
Crash Severity	Slight Crash	568	80.2	80.2
	General Crash	90	12.7	92.9
	Serious Crash	35	5.0	97.9
	Fatal Crash	15	2.1	100

By inputting actual data including crash location, crash vehicle type, rain intensity, and traffic volume into the ordered logit model (formula 10 and 11), early-warning colors can be determined by the risk matrix approach and compared with actual crash severity to validate whether the procedure of risk assessment summarized in the study is feasible. The comparison results are listed in Table 6. Among 323 crashes occurring on rainy days, totally 268 crashes are correctly predicted their warning colors, and the percentage of the correct prediction for slight, general, serious, and fatal crashes is 86.3%, 75.5%, 68.4% and 57.1%, respectively. The prediction accuracy for fatal crashes is relatively low. This could be due to the limited sample size. Moreover, Kappa and Weighted Kappa coefficients are calculated to test the consistency between early-warning colors and actual crash severities. As shown in Table 6, the two coefficients are both more than 0.6. Landis and Koch gave an interpretation of the range of Kappa coefficients: [0.6~0.8], Substantial; [0.8~1], almost perfect [23]. Considering stochastic nature of traffic accidents, the consistency between the two is fairly good and the proposed procedure in the study is able to capture actual risk implication on the freeway on rainy days.

**Table 6:** Comparisons of early-warning color and actual crash severity.

**Actual Crash Severity**	**Early-Warning Color Based on Risk Matrix**					**Correct Prediction %**
	**Blue**	**Yellow**	**Orange**	**Red**	**Total**	
Slight	214	25	5	4	248	86.3
General	5	37	7	0	49	75.5
Serious	1	2	13	3	19	68.4
Fatal	1	1	1	4	7	57.1
Total	221	65	26	11	323	83.0
**Consistency Test of Crash Severity and Early-Warning Color with SAS**						
**Coefficient**	**Value**		**ASE**		**95% Confidence Interval**	
Kappa	0.6118		0.0435		0.5265~0.6971	
Weighted Kappa	0.6474		0.0448		0.5596~0.7352	

## Discussions

### Sensitivity Analysis of Weights

A sensitivity analysis of weights was conducted to identify how early warning colors would change with weights. The original settings of weights were 0.6, 0.9, 1.2, and 1.5, for slight, general, serious and catastrophic impact, respectively. When the weights vary, the corresponding early warning colors could change. To identify such potential impact, Kappa and Weighted Kappa coefficients were calculated. As listed in Table 7, the two coefficients are much higher than 0.8. In other words, variations in the four weights will not lead to great change of early warning colors.

**Table 7:** Sensitivity analysis of four subjective weights with SAS.

Variation	Weights Setting	Kappa Coefficient	95% Confidence Interval	Weighted Kappa Coefficient	95% Confidence Interval
-0.2	(0.4, 0.7, 1.0, 1.3)	0.9596	(0.9249,0.9943)	0.9711	(0.9462,0.9960)
-0.1	(0.5, 0.8, 1.1, 1.4)	0.9596	(0.9249,0.9943)	0.9711	(0.9462,0.9960)
+0.1	(0.7, 1.0, 1.3, 1.6)	0.9425	(0.9009,0.9842)	0.9583	(0.9277,0.9889)
+0.2	(0.8, 1.1, 1.4, 1.7)	0.9342	(0.8899,0.9786)	0.9522	(0.9195,0.9849)

### Limitations

Some studies indicate that risk perception could be impacted by driver age, gender, driving experience, and accident history. However, the procedure of risk assessment summarized in the study did not analyze effects of these factors on driving risk on rainy days. Instead, these factors were considered as non-observable variables and assumed to fit a logistic distribution in the ordered logit model.

Without any doubts, the relationships among driver’s perception, behavior and actual crashes are so intricate and complicated. Even if drivers have correct perceptions of driving risk, different drivers might take various actions, resulting in different consequences. However, at least, for inexperienced drivers, this procedure could help them better identify driving risk, instead of overestimation or underestimation. In practice, due to lack of weather-related crash data, the method has been used for road weather management of some new projects in China, such as safety evaluations on the segment of National Freeway G15 and Guangshen Offshore Freeway (the Shenzhen Segment). But the improvement effects of before and after interventions would be compared and studied in the future.

In addition, visibility is not included in the model as an individual factor. If rainfall and visibility are used as two separate variables, the descriptions of rainy weather would be complicated with so many combinations. As a result, the required sample size would increase vastly. This cannot be afforded at present.

## Conclusions

According to crash statistics from the United State [1] and China [2], rainy weather has significantly negative impacts on driving safety. However, many countries and regions have not established their own warning systems to help drivers identify driving risk on rainy days due to lack of sufficient historical data. In this study, based on drivers’ risk perception, a procedure is developed for risk assessment of driving on the freeway under rainy weather conditions. The procedure includes designing a questionnaire survey to identify risk factors, building an ordered logit model to estimate impacts and corresponding probabilities, using Delphi method with focus group discussions to calculate weighted driving risk, determining four levels of early warning using a rank order cluster analysis, and finally establishing a risk matrix according to the distribution of warning colors. Furthermore, actual crash data derived from the segment of National Freeway G15 (Kilometer post: k1184+275~k1215+870) are used to validate the procedure. As a result, the proposed procedure and the risk matrix have shown the consistent results with the actual crash data. That is to say, the procedure of risk assessment in this study can help drivers identify driving risk on rainy days correctly and thus improve traffic safety.

## Supporting Information

S1 Table Drivers’ questionnaire data. (XLSX)

S2 Table Crash data from the segment of National Freeway G15. (XLSX)

S1 Text Drivers’ questionnaire form. (PDF)
