# Supplementary material for: Model Development for Risk Assessment of Driving on Freeway under Rainy Weather Conditions
Source: PLoS One. 2016 Feb 19;11(2):e0149442. doi: 10.1371/journal.pone.0149442 (PMC4764618; doi:10.1371/journal.pone.0149442)
Supplement: S1 Text — (PDF) [file pone.0149442.s003.pdf]

Driver’s Questionnaire Form

Data:____________ (mm/dd/yyyy)

Time:____________ (hh:mm)

Weather:_________ (Rain Intensity: I, II, III, IV)

Survey Location:_____________

Driver’s Gender: □ Male □ Female

Driver’s Age:_____________

Driving Experience:________(By year)

The Type of Vehicle Driven:

□ Small Vehicle □ Large Vehicle (two-axle large trucks, multi-axle large trucks)

Scenario:

You drive your owned vehicle on Freeway G15 in China under this weather condition now.

Questions:

1. Which level is perceived risk by you, if driving on the basic segment (shown in Fig. 1)?

□ Slight □ General □ Serious □ Catastrophic

2. Which level is perceived risk by you, if driving on the toll gate (shown in Fig. 1)?

□ Slight □ General □ Serious □ Catastrophic

3. Which level is perceived risk by you, if driving on the ramp (shown in Fig. 1)?

□ Slight □ General □ Serious □ Catastrophic

4. Which level is perceived risk by you, if driving on the weaving area (shown in Fig. 1)?

□ Slight □ General □ Serious □ Catastrophic

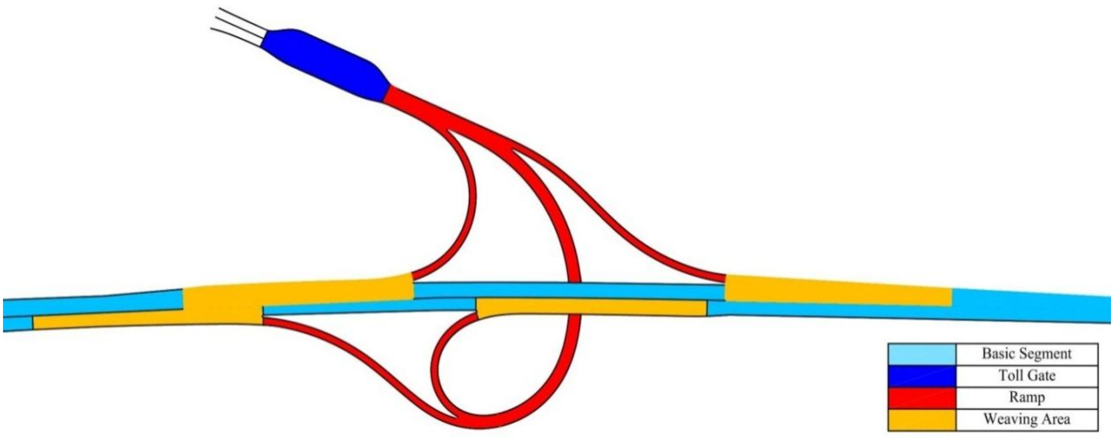

Fig. 1. Instructions for four various segments on Freeway G15.
